# Topical anti-inflammatory and anti-oxidative effects of porcine placenta extracts on 2,4-dinitrochlorobenzene-induced contact dermatitis

**DOI:** 10.1186/s12906-018-2396-1

**Published:** 2018-12-12

**Authors:** Jae Hyeok Heo, Yoonki Heo, Hee Jung Lee, Minjee Kim, Ha Youn Shin

**Affiliations:** 1 Department of Biomedical Science and Engineering, Konkuk Institute of Technology, Konkuk University, 120 Neungdong-ro, Gwangjin-gu, Seoul, 05029 South Korea; 2 Department of Biomedical-Industrial Technologies, Konkuk University, Seoul, 05029 South Korea

**Keywords:** Placenta extract, Contact dermatitis, DNCB, ROS, Hyaluronic acid, Anti-inflammatory effects

## Abstract

**Background:**

The placenta is a reservoir enriched with growth factors, hormones, cytokines and minerals. While several beneficial effects of placenta extracts on wound healing, anti-aging and anti-inflammatory responses have been reported, relatively limited mechanistic exploration has been conducted to date. Here, we provide compelling evidence of anti-inflammatory and anti-oxidative activities of porcine placenta extracts (PPE) against contact dermatitis in vivo.

**Methods:**

A contact dermatitis mouse model was established by sensitizing the dorsal skin of BALB/c mice using the contact allergen, 2,4-dinitrochlorobenzene (DNCB), and molecular consequences of topical application of PPE were investigated. PPEs were pre-sterilized via γ-irradiation, which is a milder but more effective way of sterilizing biomolecules relative to the conventional autoclaving method.

**Results:**

DNCB-induced skin lesions displayed clear contact dermatitis-like symptoms and topical application of PPE dramatically alleviated both local and systemic inflammatory responses. Inflammatory epidermal thickening was completely abrogated and allergen-specific serum IgE levels significantly reduced in the presence of PPE. Moreover, anti-oxidative activities of PPE were observed both in vitro and in vivo, which may lead to attenuation of inflammatory responses. Prolonged treatment with PPE strongly inhibited production of DNCB-induced reactive oxygen species (ROS) and subsequently prevented oxidative degradation of hyaluronic acid (HA), which triggers innate inflammatory responses.

**Conclusion:**

Our findings supply valuable insights into the mechanisms underlying the anti-inflammatory effects of PPE and provide a functional basis for the clinical application of PPE in inflammatory diseases.

**Electronic supplementary material:**

The online version of this article (10.1186/s12906-018-2396-1) contains supplementary material, which is available to authorized users.

## Background

The placenta is a temporary organ in female mammals during gestation that connects the fetus to the mother’s uterine wall, providing oxygen, nutrients and fetal immunity to the developing fetus. The placenta serves a plethora of growth factors, hormones, minerals, vitamins and cytokines, and also provides uracil, tyrosine, phenylalanine and tryptophan that control oxidative stress [1–3]. Several studies to date have attempted to utilize placenta extracts for medical treatment purposes or as a cosmetic ingredient. The clinical efficacy of placenta extracts has been widely investigated and their activity in wound healing and anti-aging established [1, 4, 5]. Additionally, anti-inflammatory and anti-oxidative effects of human placenta extracts have been reported [2, 6, 7]. While the beneficial effects of human placenta extracts in relieving inflammatory skin disease have been widely investigated [8–10], little is known about the underlying molecular mechanisms.

One of the common inflammatory skin diseases is ‘contact dermatitis’ triggered by contact with foreign substances, such as natural or synthetic chemicals. These types of foreign molecules elicit allergies or skin irritations resulting in inflammatory symptoms, such as itchiness, red rash, blisters and burning skin. Contact dermatitis is clinically subdivided into two distinct types: irritant contact dermatitis (ICD) and allergic contact dermatitis (ACD) [11, 12]. ICD is triggered by direct contact with toxic chemicals, such as solvents, latexes, and cosmetics. ACD is the more prevalent form triggered by low molecular weight allergens (< 500 Da), such as nickel, fragrances, dyes and preservatives.

In contrast to ICD, which induces instant local inflammation, ACD predominantly induces adapted immunity mediated by T or B cells or a subset of natural killer (NK) cells. ACD is triggered via two steps comprising a sensitization phase and an elicitation phase [11, 13]. In the sensitization phase, the contact allergen is recognized as a foreign substance by antigen presenting cells (APC), for instance, Langerhans cells or dermal dendritic cells. Stimulated APCs migrate to local lymph nodes where they present the contact allergen to naïve T cells, leading to the generation of allergen-specific effector T cells. In the elicitation phase, re-exposure to the same allergen triggers recruitment of neutrophils, monocytes and effector T cells to inflammation regions, inducing secondary immune responses.

Recent studies on human cells or the mouse model for ACD have demonstrated that contact allergens also induce oxidative stress via production of ROS [14–16]. ROS depolymerize a component of the extracellular matrix (ECM) known as HA and further promote expression of hyaluronidase, an enzyme that can break down high molecular weight HA to a lower molecular weight. Fragmented HA is recognized as an endogenous danger signal to pattern recognition receptors (PRR), such as Toll-like receptors (TLR). Low molecular weight HA activates TLR2 and TLR4, consequently eliciting pro-inflammatory innate immune responses [16].

A previous report by our group demonstrated anti-inflammatory activity of PPE in vitro [7]. Treatment of the lipopolysaccharide-stimulated murine macrophage cell line, RAW 264.7, with γ-irradiated PPE led to a dramatic reduction in nitric oxide and pro-inflammatory cytokine levels. In the present study, we further investigated the anti-inflammatory and anti-oxidative activities of PPE in vivo and the underlying molecular mechanisms using the contact dermatitis mouse model.

## Methods

### Experimental mice

Female BALB/c mice were purchased from Orient Bio (Seongnam-si, Korea) and maintained in individual cages under specific pathogen-free conditions at 22 ± 1 °C with 12 h of light/dark cycle. Six-week-old mice with an average body weight of 18 g were employed for the study and euthanized by CO_2_ inhalation after the termination of each experiment. All animal procedures were approved by the Institutional Animal Care and Use Committee (IACUC) of Konkuk University.

### Preparation of PPE

Placenta extracts were isolated from pig placenta, kindly provided by KR Biotech (Seoul, Korea). Pig placenta were obtained immediately after vaginal deliveries, and the umbilical cord and amnion were discarded. Remaining tissue was exhaustively washed with ice-cold phosphate buffered saline (PBS) to remove all traces of blood. Porcine placenta tissues were disrupted using a tissue homogenizer (Tissue Tearor, Biospec Products Inc., Bartlesville, OK, USA) in cold PBS. Tissue homogenates were centrifuged at 6000 g for 15 min, and the supernatants were lyophilized. Sterilization of placenta extracts was performed via γ-irradiation in air using an isotope source of Cobalt-60 at Greenpia Technology Co. (Seoul, Korea). The absorbed γ-irradiation dose was 25 kGy at a rate of 1 kGy/h.

### Induction of contact dermatitis with a DNCB sensitizer and topical PPE treatment

One day prior to sensitization, mice dorsal hairs were completely shaved with an electric trimmer and depilatory cream applied (total 30 mice). For sensitization, 0.7 cm^2^ gauze-attached patches (Tegaderm™, 3M™ Health Care, St. Paul, MN, USA) were treated with 150 μl of 1% DNCB and applied to the back skin of mice for 20 h on days 0, 3, and 6. For elicitation of responses, patches were treated with 100 μl of 0.5% DNCB and applied to back skin of mice for 20 h on days 9, 12, and 15. DNCB (99% purity, Sigma-Aldrich, St. Louis, MO, USA) was resolved in Acetone–olive oil (AOO) (Sigma Aldrich, St. Louis, MO, USA). Mice were randomly divided into 5 groups representing different experimental conditions (*n* = 6 per group). To examine the potential beneficial effects of PPE on contact dermatitis, back skin of mice were topically treated with PPE or dexamethasone (DEX, a positive control) on a daily basis after removal of DNCB patches. PPE (10 mg) was thoroughly mixed with 1 g of base cream and 3 mg DEX (0.3 g daily) with 1 kg of base cream using a homogenizer. PPE or DEX cream mixtures were applied to back skin of mice with until the end of the experiment (day 17). Mice were treated with cream alone as a negative control group to determine the effect of vehicle itself on inflammatory responses.

### Histopathological and immunohistochemical (IHC) analyses

Back skin tissues of mice (1 cm^2^) were collected, fixed with neutral buffered formalin (NBF) for 24 h, and embedded in paraffin. Paraffin blocks were cut into 3 μM thick sections and stained with hematoxylin and eosin (H&E) using a standard protocol (Abion CRO, Seoul, Korea). Epidermal thickness was measured at the thickest part of five different dorsal epidermis specimens per mouse group, and significant differences between each pair of conditions were analyzed.

### Measurement of DNCB-induced serum IgE and IgG

DNCB-induced serum IgE and IgG levels were assessed using an enzyme-linked immunosorbent assay (ELISA) kit. Briefly, blood samples of mice were obtained from facial veins on the last day of the experiment (day 17) (*n* = 3 per group). Collected samples were incubated at room temperature for 30 min and centrifuged at 3500 rpm for 25 min, and supernatants were stored at − 80 °C until use. The 96-well plates were pre-coated with 0.17 mg/ml DNP-ovalbumin (Santa Cruz, Dallas, TX, USA), and then blocked with 2% BSA for 1 h at 37 °C, followed by washing with PBS-T. Diluted serum samples and standards were added to each well and incubated for 2 h at room temperature. After washing with PBS-T, goat anti-mouse IgG (Santa Cruz, Dallas, TX, USA) and goat anti-mouse IgE (Southern Biotech, Birmingham, AL, USA) in 1% PBS were added at ratios of 1:4000 and 1:2000, respectively. Following several wash steps, TMB Peroxidase EIA Substrate (Bio-Rad, Hercules, CA, USA) was added to each well for colorimetric development, and the reaction was subsequently terminated with 1 N H_2_SO_4_. Absorbance was detected at 450 nm using a microplate reader (Epoch Microplate Spectrophotometer, BioTek, Winooski, VT, USA).

### Cell culture

The human keratinocyte cell line, HaCaT, was obtained from the Korean Cell Line Bank (Seoul, Korea). Cells were grown in Dulbecco’s modified Eagle’s medium (Thermo Fisher Scientific, Waltham, MA, USA) supplemented with 10% fetal bovine serum (FBS) and 1% penicillin and streptomycin.

### Cellular ROS detection assay using H_2_DCFDA

The DNCB-induced total ROS content in HaCaT cells was measured using 2′,7′-dichlorofluorescein diacetate (H_2_DCFDA), a ROS detection reagent (Thermo Fisher Scientific, Waltham, MA, USA). H_2_DCFDA, a cell-permeable non-fluorescent probe, is oxidized into a highly fluorescent compound in cells. Intracellular fluorescence intensity is proportional to the amount of ROS generated in cells. Prior to the experiment, HaCaT cells were pre-incubated with serum-free DMEM containing PPE or DEX for 24 h. Incubation medium was collected and stored at − 20 °C until use. For the ROS detection assay, HaCaT cells were plated on 96-well plates (Sigma Aldrich, St. Louis, MO, USA) at a density of 2 × 10^4^ cells per well. The next day, PPE or DEX pre-incubated medium was gently mixed with 40 μM DNCB-treated medium, added to each well, and incubated for different time periods (3, 6, 12, 24, and 48 h). After washing with PBS, cells were treated with 5 μM H_2_DCFDA for 30 min at 37 °C in the dark and cell nuclei were counterstained with 4′,6-diamidino-2-phenylindole (DAPI) in mounting solution for 30 min at RT. Following several washes, DCF fluorescence and DAPI staining were visualized under a fluorescence microscope (Eclipse Ti, Nikon, Tokyo, Japan), and the representative images were captured using a NIS-Elements image browser.

### Ex vivo ROS detection assay using dihydroethidium (DHE)

Ears of mice were topically pre-treated with PPE or DEX mixed with cream for 15 min and subsequently irritated with 5% DNCB to trigger ROS production. After 15 min, mice were euthanized and 0.5 cm^2^ ear sections were obtained for the ROS detection assay. Ear sections were incubated ex vivo with 5 mM DHE (Sigma Aldrich, St. Louis, MO, USA) in DMSO for 30 min in the dark. DHE is an ethidium-based fluorescent dye that intercalates within DNA of cells upon oxidization, staining the nucleus a bright fluorescent red. After washing with PBS, ear tissues were placed on glass slides and ROS production were monitored by visualization under a fluorescence microscope (Eclipse Ti, Nikon, Tokyo, Japan).

### Statistical analysis

Significant differences between pairs of groups were analyzed by two-tailed Student’s *t*-tests or one-way ANOVA followed by Tukey’s multiple comparisons tests. *P* < 0.05 was considered statistically significant.

## Results

### Topical PPE application attenuates DNCB-induced inflammatory responses in mice

**Fig. 1:**
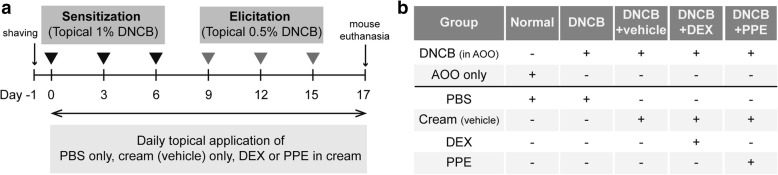
Schematic diagram of the experimental design. (**a**) Induction of contact dermatitis in mice and topical PPE treatment of skin lesions. Skin inflammation was induced on the back skin of BALB/c mice by periodic topical application of DNCB-treated patches. One day prior to sensitization, mouse dorsal hairs were completely removed, and 1% DNCB-treated patches were attached to back skin for 20 h on days 0, 3 and 6. Following sensitization, 0.5% DNCB patches were applied on days 9, 12, and 15 for elicitation of responses. Upon removal of DNCB patches, PPE in cream was topically applied to skin lesions every day until the end of the experiment (total of 17 days). PBS or cream (vehicle) alone was applied as a negative control and DEX treatment used as a positive control. (**b**) Experimental conditions for each mouse group. Mice were divided into 5 groups representing different experimental conditions (*n* = 6 per group). Except for the normal group, DNCB in AOO was applied to four mouse groups (designated DNCB, DNCB+vehicle, DNCB+DEX, DNCB+PPE) to induce skin inflammation. PPE was mixed with cream and topically applied to skin lesions to determine effects on inflammation

Organic compounds, such as DNCB, are commonly used as sensitizers to mimic contact dermatitis-like symptoms in mice. To evaluate the anti-inflammatory effects of PPE in vivo, we initially established a contact dermatitis mouse model using DNCB as the stimulating agent. Shaved mouse back skin was repeatedly irritated with DNCB-treated patches, and subsequent inflammatory responses were monitored. For initial sensitization, 1% DNCB-treated patches were attached to back skin of mice three times a week with 3-day intervals (days 0, 3, and 6) and challenged with 0.5% DNCB-treated patches three times with equivalent time intervals (days 9, 12, 15). To examine anti-inflammatory effects, PPE was mixed with cream (vehicle) and topically applied to skin lesions daily for 17 days (Fig. 1a). Mice treated with DEX mixed with cream were used as the positive control and those administered cream alone or PBS as the negative control (Fig. 1b).

**Fig. 2:**
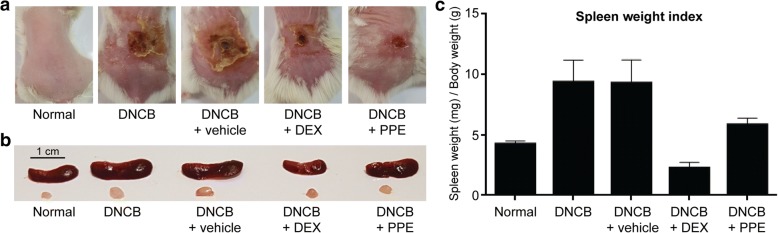
Establishment of a DNCB-induced contact dermatitis mouse model and evaluation of the anti-inflammatory effect of PPE. (**a**) Representative images depicting contact dermatitis-like symptoms. Repeated application of DNCB-treated patches on back skin of mice was sufficient to trigger skin inflammation resembling contact dermatitis. Co-treatment with PPE or DEX led to a significant reduction in the clinical severity. (**b**) Size comparison of spleen (upper panels) and draining lymph node (lower panels) with or without PPE treatment. Spleens and lymph nodes were isolated from all mouse groups on day 17. Treatment with DNCB alone or DNCB in vehicle (cream) led to an increase in both spleen and lymph node sizes. Application of either PPE or DEX inhibited enlargement of these immune organs. (**c**) Comparison of spleen weights as an inflammation index. The spleen weight of each mouse group was normalized to body weight (*n* = 3 for each group)

In the sensitization phase, DNCB-treated mice started to show contact dermatitis-like symptoms, including red rashes, scales, blisters, and scabs. All groups displayed similar clinical symptoms, except the control (normal) group. Upon repeated exposure to contact allergens, inflammatory responses became severe and all groups showed skin lesions after 10 days, with the severity of the lesions depending on the type of daily topical treatment. Notably, topical application of PPE or DEX led to a prominent reduction in the area of skin lesions whereas cream alone had no effect (Fig. 2a). A common feature of chronic inflammation is enlargement of immune organs including spleen and lymph nodes [8, 17]. Consistently, both spleen and lymph nodes isolated from DNCB-treated mice were markedly enlarged, compared to those of the normal group (Fig. 2b). Co-treatment with PPE or DEX clearly suppressed this enlargement of spleen and lymph nodes, resulting in sizes comparable to those of the normal group. In addition to size, spleen weights were compared between mouse groups for evaluating the anti-inflammatory effects of PPE. After DNCB stimulation, the spleen weight index increased up to 2.2-fold, compared to the normal group (Fig. 2c). However, topical PPE application reduced the weight index by 60% relative to DNCB-treated mice, which was statistically comparable to the control group. Our data indicate that PPE is capable of attenuating both local and systemic physical inflammatory reactions elicited by DNCB.

### PPE treatment alleviates epidermal hyperplasia and reduces serum IgE levels

**Fig. 3:**
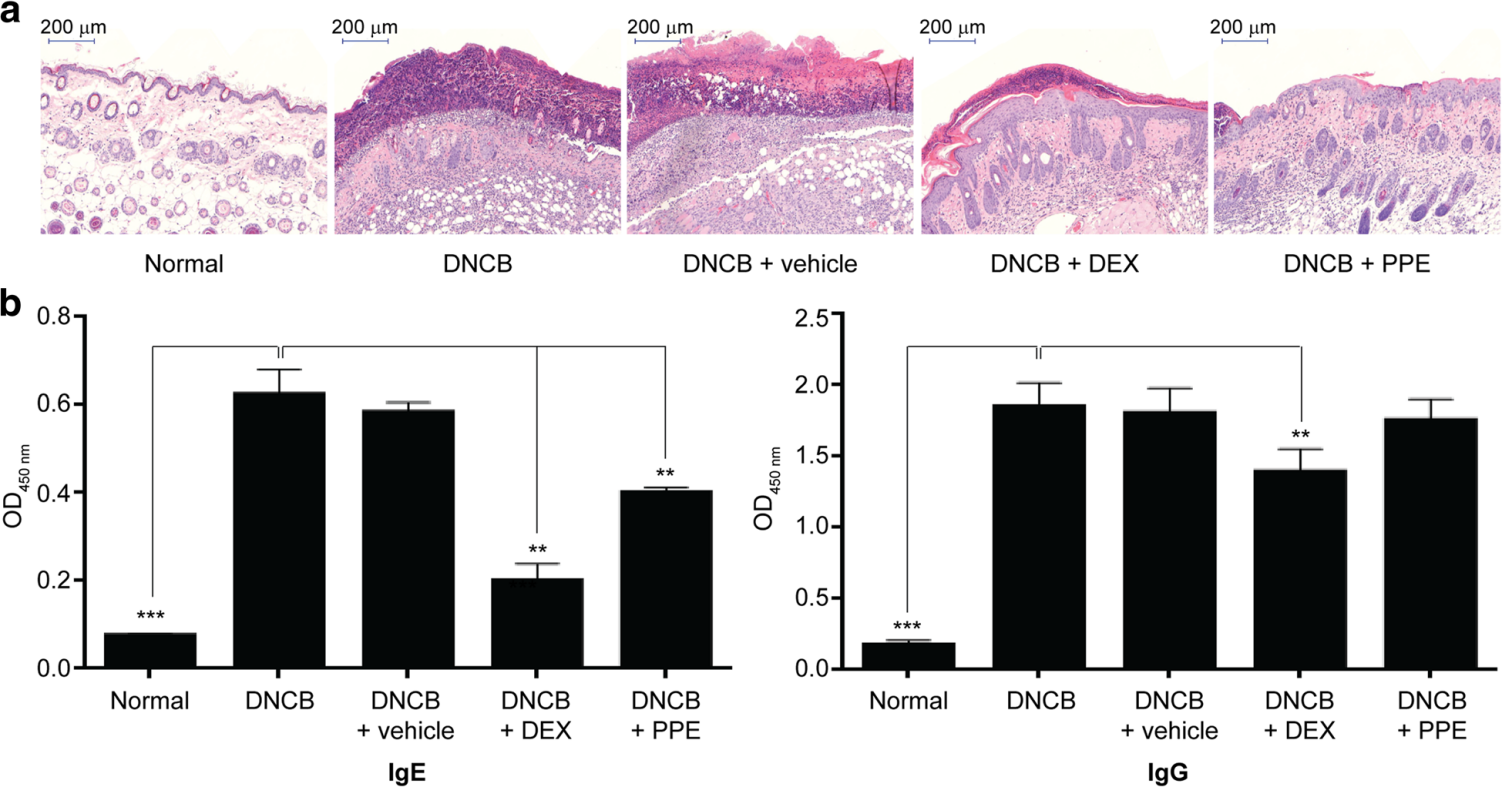
Inhibitory effect of PPE on DNCB-induced inflammation based on histological analyses and serum IgE levels. (**a**) Histopathological changes in mouse dorsal inflammatory lesions were analyzed via H&E staining. Abnormal thickening of the epidermis along with severe keratinization and massive infiltration of immune cells were observed in DNCB-treated mice. Co-treatment with PPE significantly suppressed epidermal hyperplasia whereas cream alone had no effect. DEX treatment was used as a positive control. (**b**) IgE and IgG levels. Serum IgE and IgG contents were measured using ELISA kits. Both IgE and IgG levels were significantly enhanced (up to 9-fold) following DNCB treatment. Only allergen-specific IgE levels were significantly reduced after PPE treatment whereas IgG levels were maintained. Cream (vehicle) alone had no suppressive effect on either IgE or IgG levels. Results are presented as means ± SD of independent biological replicates (*n* = 3). The two-tailed student *t*-test was used to evaluate the statistical significance of differences between DNCB-treated and normal, PPE or DEX-treated groups (** *P* < 0.01, *** *P* < 0.001)

One of the histological characteristics of contact dermatitis is thickening of the epidermis and outer dermis along with infiltration of immune cells [8, 17]. To further evaluate the anti-inflammatory effect of PPE on skin lesions, histopathological alterations were investigated. On the last day of the experiment (day 17, Fig. 1a), mice were euthanized, and then back skin displaying inflammatory lesions were stained with H&E for histological analyses. As expected, skin sections collected from DNCB-treated mice revealed abnormal thickening of epidermis and outermost layer of the dermis, resulting from epidermal hyperplasia as well as severe keratinization (including hyperkeratosis and parakeratosis). In addition, significant vasodilation and increased immune cell infiltration in the dermis were evident (Fig. 3a, Additional file 1: Figure S1). Administration of PPE or DEX led to remarkable attenuation of the histological changes induced by DNCB. Suppression of epidermal hyperplasia by PPE was substantial and the resulting histological appearance of skin was comparable to that of the normal group. Since treatment with cream alone (vehicle) did not improve histopathological symptoms, we conclude that the anti-inflammatory effect is solely attributable to PPE components.

DNCB sensitization is commonly employed as a method to elicit cell-mediated immunity [17–21]. Recent reports have shown that DCNB is dispensable for inducing humoral immune responses through type I hypersensitivity, which produces allergen-specific antibodies [8, 22, 23]. To further establish the suppressive effects of PPE on immune responses, serum immunoglobulin levels were measured using ELISA and compared between each mouse group. Allergen-specific serum IgE levels were significantly increased in mice treated with DNCB alone (~ 6-fold), compared to the normal group (Fig. 3b, Additional file 1: Figure S2). Administration of PPE resulted in a significant decrease in serum IgE levels by 60% relative to the DNCB-treated group, while cream (vehicle) alone did not exert an apparent effect. Serum IgG levels were additionally elevated in response to DNCB treatment but remained unaffected by PPE. Our findings support anti-inflammatory effects of PPE on allergen-specific immune responses.

### PPE abrogates DNCB-induced ROS production in vitro and in vivo

**Fig. 4:**
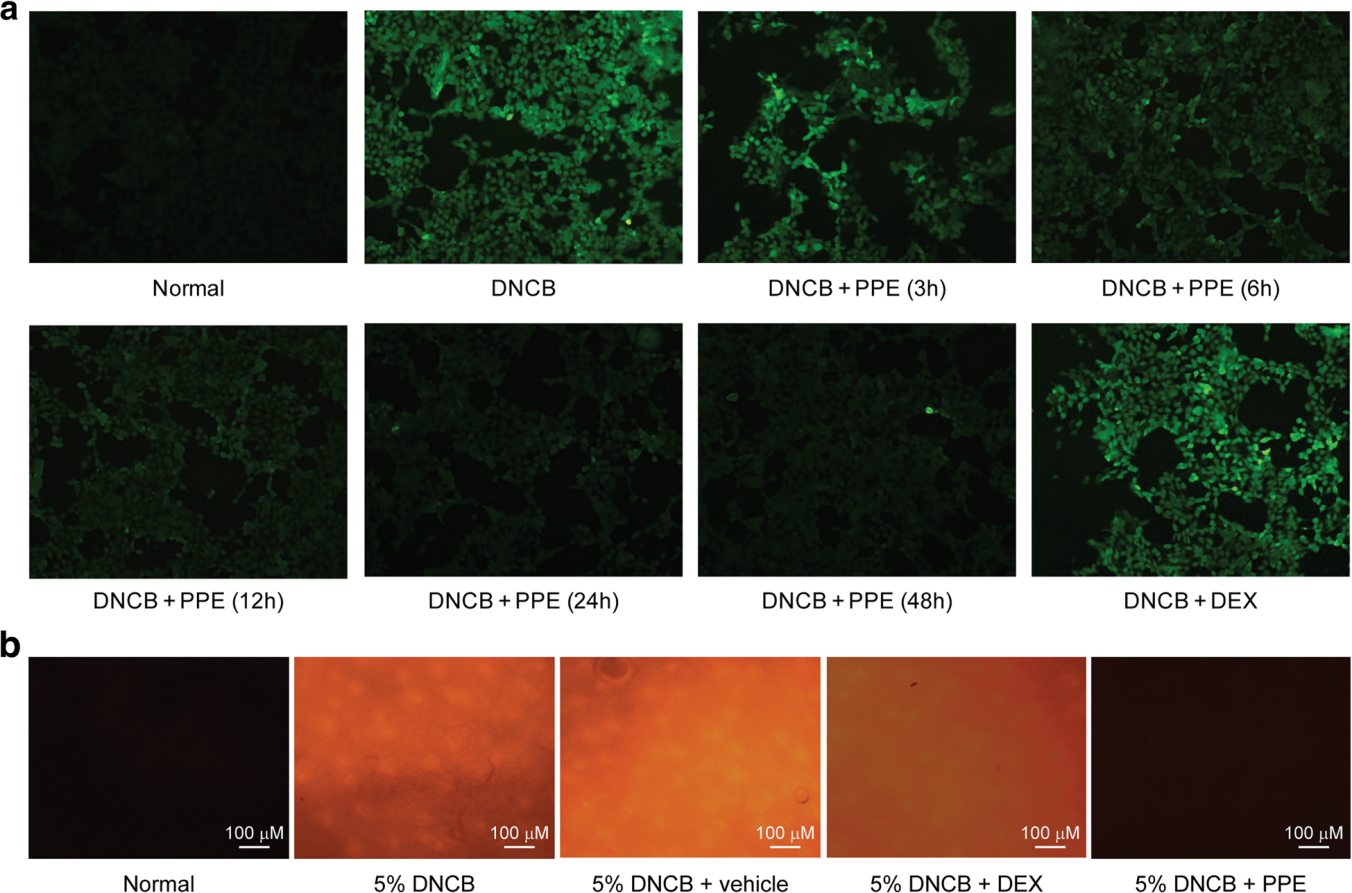
In vitro and in vivo anti-oxidative activity of PPE on DNCB-induced ROS. (**a**) Cellular ROS production was induced by 40 μM DNCB in HaCaT cells. To determine the effect of PPE on DNCB-induced ROS production, HaCaT cells were co-treated with DNCB and PPE for different time periods (3, 6, 12, 24 and 48 h). Intracellular ROS were stained with the H_2_DCFDA probe, and the fluorescence was visualized under a microscope. DNCB generated significant amounts of ROS in cells, which gradually disappeared with PPE treatment. After 48 h of PPE treatment, fluorescence intensity diminished to the basal level (normal group). DEX did not display anti-oxidative activity after 24 h of treatment. (**b**) Inhibitory effect of PPE on ROS generation in vivo. Ears of mice were pre-treated with cream (vehicle) alone, PPE or DEX in cream for 1 h before induction of ROS by topical application of 5% DNCB. Normal and 5% DNCB-treated groups were treated with PBS only or PBS with DNCB. Ear tissues were obtained after mouse euthanasia and stained ex vivo with 5 mM DHE in DMSO for 30 min. Fluorescence generated by ROS was visualized under a microscope. DNCB induced significant ROS production, which was almost completely abrogated by PPE

Continuous exposure to contact allergens triggers ROS production leading to cellular damage [14]. To ascertain whether the anti-inflammatory activity of PPE is exerted through inhibition of ROS production, ROS were generated in HaCaT cells via DNCB treatment and intracellular ROS levels were evaluated using the H_2_DCFDA probe, a non-fluorescent compound that is converted to a fluorescent molecule upon reaction with oxygen species. The majority of HaCaT cells treated with DNCB showed strong fluorescence, indicative of excessive ROS production (Fig. 4a, Additional file 1: Figure S3). Co-treatment with PPE led to a gradual decrease in cellular ROS levels. Cellular fluorescence started to decrease after 3 h of PPE treatment and intensity was clearly diminished after 12 h. Within 48 h of PPE treatment, cellular fluorescence was barely detectable, which was similar to the basal level. Our results indicate that PPE exerts anti-oxidative effects and prolonged treatment promotes ROS scavenging activity.

To assess the anti-oxidative activity of PPE in vivo, PPE, DEX or cream alone was administered to ears of mice, and ROS production was induced via topical application of DNCB. ROS produced by DNCB treatment was stained with DHE fluorescent dye ex vivo after mice were sacrificed. Ear sections stimulated with DNCB exhibited strong fluorescence relative to the normal group, indicative of substantial ROS production (Fig. 4b). Cream alone did not exert a noticeable effect on ROS generation while DEX exerted a modest inhibitory effect. Conversely, PPE blocked ROS production significantly in vivo and completely suppressed fluorescence. Data from both in vitro and in vivo studies collectively demonstrate that PPE exerts strong anti-oxidative effects by suppressing DNCB-induced ROS production.

### PPE inhibits ROS-induced HA degradation

Contact sensitizers induce skin inflammation through ROS production with concomitant breakdown of the ECM component, HA [16, 24]. To ascertain whether PPE suppresses DNCB-induced ROS production and skin inflammation, we examined its effect on ROS-induced HA degradation. We employed the Cu(II)/SO_4_/H_2_O_2_ system commonly used to generate ROS in vitro [16] as a source of ROS production. ROS-inducing compounds were mixed with high molecular weight HA with or without PPE. Combination of CuSO_4_ with 100 mM or 200 mM H_2_O_2_ resulted in clear HA fragmentation while CuSO_4_ or H_2_O_2_ alone had no effect (Additional file 1: Figure S4a). DEX did not exert an inhibitory effect on HA degradation. In contrast, both 0.1 and 1% PPE unequivocally blocked ROS-mediated HA fragmentation.

HA can be degraded not only through non-enzymatic reactions but also a family of enzymes known as hyaluronidases [16, 25, 26]. The potential involvement of PPE in hyaluronidase activity was evaluated using zymography. Briefly, back skin tissues were isolated from DNCB-sensitized mice, and hyaluronidase activity was measured in tissue lysates. DNCB treatment enhanced hyaluronidase activity by ~ 1.5-fold, compared to the normal group (Additional file 1: Figure S4b), which was not affected by either cream or DEX alone. However, PPE treatment clearly reduced hyaluronidase activity by ~ 30% relative to that in DNCB-treated mice, which was even lower than that of the normal control group. Our results support the theory that PPE prevents HA degradation by suppressing hyaluronidase activity.

## Discussion

While placenta extracts are reported to exert multiple beneficial effects on wound healing, anti-aging and anti-inflammatory responses, their in vivo efficacy in animal models and underlying molecular mechanisms remain to be established. Data from the current study present strong evidence that γ-irradiated PPE exerts anti-inflammatory and anti-oxidative effects in a contact dermatitis mouse model in vivo. Placental sterilization is an essential step in removing infectious agents to ensure the safety of PPE application for clinical use. The conventional method of sterilization is simple autoclaving [7, 8]. However, since autoclaving employs extremely high temperatures and pressures to sterilize microbes, the process may simultaneously inactivate beneficial bioactive peptides present in placental extracts in addition to killing pathogens. To protect the activities of potent placenta components, we used a milder sterilization method of γ-irradiation. This technique has been used to purify various medical sources and the standard protocol and intensity of irradiation for clinical purposes are well established [27]. Previous studies by our group demonstrated anti-inflammatory effects of γ-irradiated PPE in vitro*.* Here, we provide evidence of anti-inflammatory and anti-oxidative activities of PPE in vivo [7].

DNCB is a cycloheximide benzene commonly used to trigger both innate and adaptive immunity that mimics contact dermatitis-like symptoms in mice [17, 22, 23, 28, 29]. Upon repeated application of DNCB-treated patches to mouse dorsal skin, contact dermatitis-like symptoms became evident, including red rashes, scabs and edema of immune organs. Topical application of PPE or DEX relieved inflammation of skin and prevented the increase in size and weight of immune organs, including lymph nodes and spleen. Notably, topical PPE treatment had a significantly stronger alleviatory effect on epidermal hyperplasia and hyperkeratosis in DNCB-induced inflammatory skin lesions than DEX.

Although both PPE and DEX exerted anti-inflammatory effects in DNCB-induced contact dermatitis mice, we observed a number of differences between their actions. For example, whereas PPE specifically reduced allergen-specific IgE, DEX induced reductions in both IgE and IgG levels and was slightly better at reducing inflamed spleen size. PPE also showed anti-oxidative activity against DNCB-induced ROS generation. Notably, prolonged treatment with PPE strongly inhibited ROS generation by DNCB in both HaCaT cells and a mouse model of contact dermatitis, whereas DEX did not prevent ROS production, either in vitro or in vivo. These findings suggested that the anti-inflammatory activities of PPE and DEX are regulated via slightly different mechanisms, which require further elucidation in future studies.

To further clarify the mechanisms underlying the anti-oxidative activity of PPE, we investigated its potential involvement in oxidative degradation of HA, a major component of the ECM that exists as high molecular mass polymers (> 10^6^ Da). High molecular weight HA exerts anti-inflammatory, anti-angiogenic and immunosuppressive activities while low molecular weight HA (< 50 Da) elicits an opposite response [30]. Fragmented HA is recognized by TLR2 and TLR4 and induces proinflammatory responses [31, 32]. In our experiments, pre-treatment with PPE clearly inhibited HA degradation in the Cu(II)/H_2_O_2_ system whereas DEX had no effect, in accordance with the results of the ROS scavenging assay. We have previously identified major active components in the aqueous fraction of PPE, including α-fetoprotein precursor, prefoldin subunit 5, heat shock factor protein 4, vimentin, lebercilin-like protein, precursors of cholecystokinins and lung and nasal epithelium carcinoma-associated protein 1, glycogen phosphorylase, odorant binding proteins, and vinculin [33]. Our previous study also suggested that α-fetoprotein may act as a major anti-oxidant factor. To obtain reproducible results, we used consistent levels of active PPE components for each experiment. We measured the total protein amount of PPE using a protein quantification assay and generated each PPE batch with the same protein concentration. Although it would be ideal to measure the concentration of individual active PPE components or a single key component, such as α-fetoprotein, to evaluate the consistency of each batch, it is technically challenging to measure the levels of every component each time and would not be appropriate to select a single component as a standard molecule because PPE is a mixture of many different kinds of active components having both known and unknown functions. Based on the collective data, we propose that PPE alleviates inflammatory skin disease through two distinct routes: (1) preventing cellular and humoral immunity and (2) inhibition of innate inflammatory responses through prevention of oxidative stress. Further research is warranted to determine whether these distinct routes act synergistically or independently.

Contact dermatitis can generally be prevented by avoiding allergens and irritants but complete isolation from all environmental chemicals is not possible. Topical application of Calamine lotion or barrier cream is used to relieve itchiness or mild symptoms to a slight extent. However, in cases where symptoms do not improve and itches and pains become severe, corticosteroids (such as hydrocortisone and dexamethasone) should be prescribed by dermatologists. Unfortunately, prolonged treatment with steroids associated with various side-effects. Data from the current study suggest that PPE has distinct anti-inflammatory and stronger anti-oxidative activities compared to DEX. The synergistic anti-inflammatory and anti-oxidative effects of PPE may be effectively utilized to develop potent clinical therapeutics for contact dermatitis.

## Conclusions

Topical treatment of γ-irradiated porcine placenta extracts on the skin lesion dramatically reduced both local and systemic inflammatory responses. Moreover, it also showed the anti-oxidative activity by inhibiting the generation of reactive oxygen species, which results in the prevention of the hyaluronic acids degradation that can trigger inflammatory responses. Our findings provide the fundamental understanding of the action of placenta extracts and further provide a new insight into using placental extracts on the treatment of inflammatory skin diseases.

## Additional file

Additional file 1: **Figure S1.** PPE dramatically alleviated epidermal thickening of DNCB-induced inflammatory lesions in vivo; **Figure S2.** IgE and IgG levels; **Figure S3.** Nuclear staining in HaCaT cells; **Figure S4.** PPE prevents the oxidative degradation of HA by inhibiting hyaluronidase activity; Supplementary methods: Detection of ROS-induced HA degradation in vitro and HA zymography. (DOCX 1687 kb)

## Abbreviations

ACD: Allergic contact dermatitis
AOO: Acetone–olive oil
APC: Antigen presenting cell
DEX: Dexamethasone
DHE: Dihydroethidium
DNCB: 2,4-dinitrochlorobenzene
ECM: Extracellular matrix
ELISA: Enzyme-linked immunosorbent assay
FBS: Fetal bovine serum
H&E: Hematoxylin and eosin
H_2_DCFDA: 2′,7′-dichlorofluorescein diacetate
HA: Hyaluronic acid
IACUC: Institutional animal care and use committee
ICD: Irritant contact dermatitis
IHC: Histopathological and immunohistochemical
NBF: Neutral buffered formalin
NK: Nature killer
PBS: Phosphate buffered saline
PPE: Porcine placenta extract
PRR: Pattern recognition receptor
ROS: Reactive oxygen species
TLR: Toll-like receptor
