# Supplementary material for: Topical anti-inflammatory and anti-oxidative effects of porcine placenta extracts on 2,4-dinitrochlorobenzene-induced contact dermatitis
Source: BMC Complement Altern Med. 2018 Dec 12;18:331. doi: 10.1186/s12906-018-2396-1 (PMC6291973; doi:10.1186/s12906-018-2396-1)
Supplement: Supplementary file 1 — Figure S1. PPE dramatically alleviated epidermal thickening of DNCB-induced inflammatory lesions in vivo; Figure S2. IgE and IgG levels; Figure S3. Nuclear staining in HaCaT cells; Figure S4. PPE prevents the oxidative degradation of HA by inhibiting hyaluronidase activity; Supplementary methods: Detection of ROS-induced HA degradation in vitro and HA zymography. (DOCX 1687 kb) [file 12906_2018_2396_MOESM1_ESM.docx]

**Additional file 1**

1. Supplementary Figures
2. Supplementary Methods

**
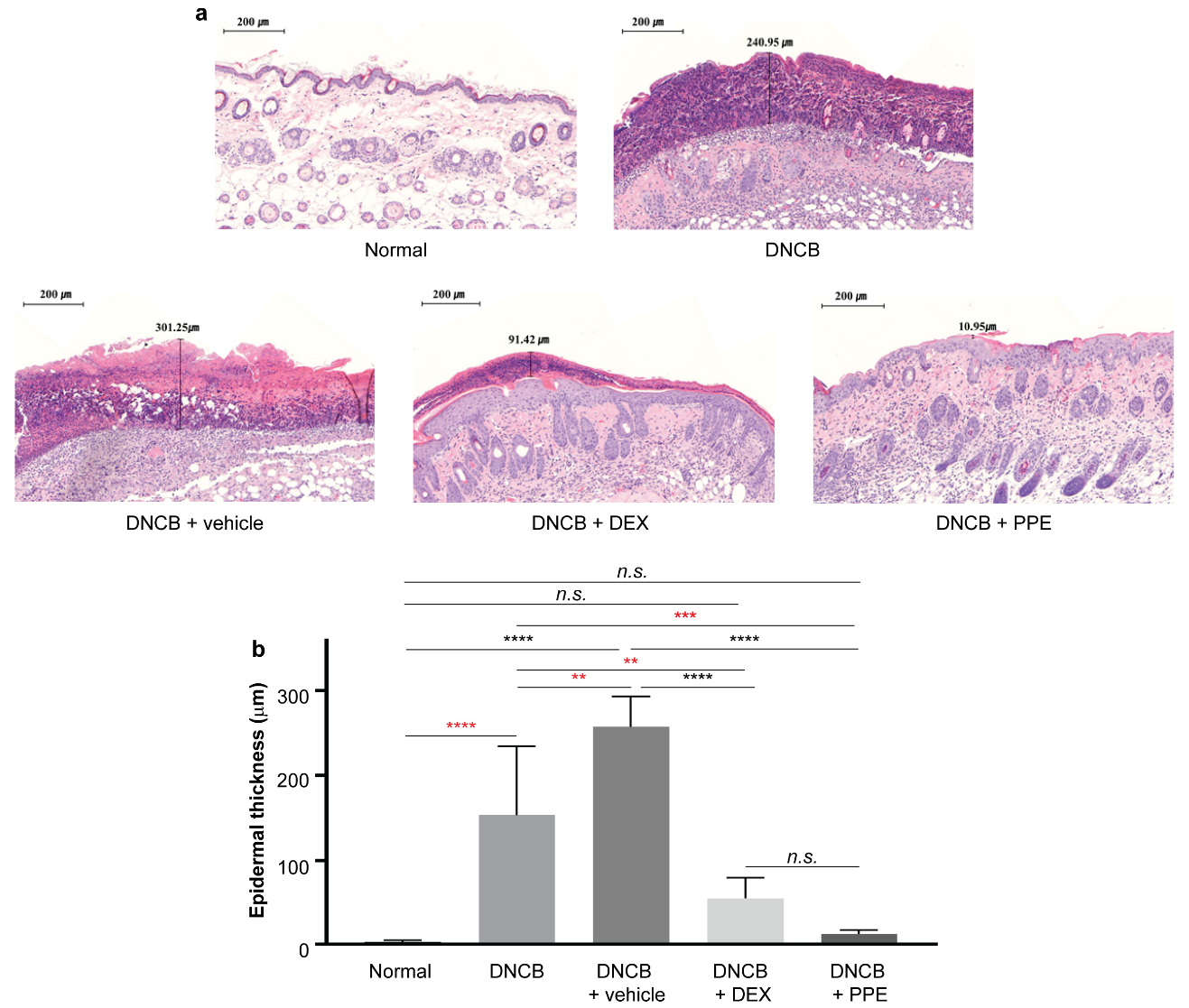
**

**Figure S1** PPE dramatically alleviated epidermal thickening of DNCB-induced inflammatory lesions *in vivo*. (a) Representative histological images showing the anti-inflammatory effects of PPE on epidermal thickening. Black lines indicate the thickness (μm) of mouse dorsal epidermis. (b) Five different sections of dorsal epidermis were obtained from each mouse group and histological changes were analyzed by H&E staining. Results are presented as mean ± SD of five replicates. The difference in epidermal thickness between mice treated with DNCB alone and mice treated with DNCB and other factors (indicated by red asterisks) or between pairs of groups (black asterisks) were analyzed by one-way ANOVA followed by Tukey’s multiple comparisons test. * *P* < 0.05, ** *P* < 0.01, *** *P* < 0.001, **** *P* < 0.0001, *n.s.* not significant.

**
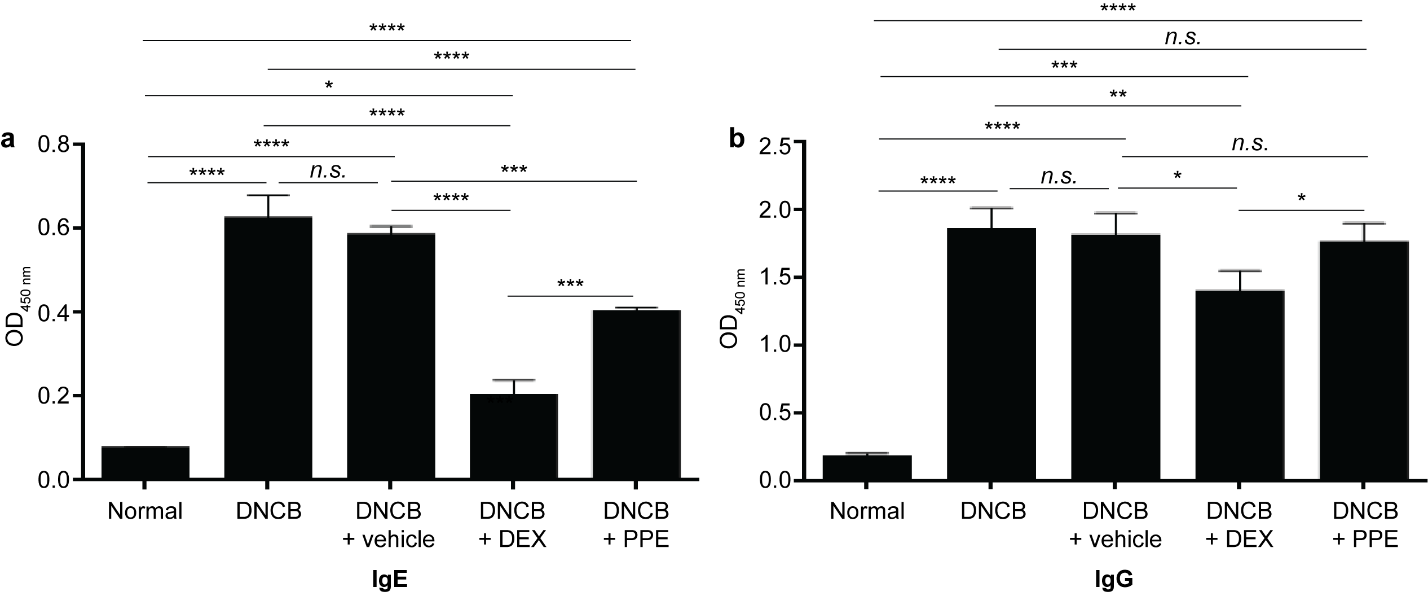
**

**Figure S2** IgE and IgG levels. (a) Serum IgE and (b) IgG levels were measured by ELISA. Results are presented as mean ± SD of independent biological replicates (*n* = 3). Significant differences between pairs of groups were analyzed by one-way ANOVA followed by Tukey’s multiple comparisons test. * *P* < 0.05, ** *P* < 0.01, *** *P* < 0.001, **** *P* < 0.0001, *n.s.* not significant.

**
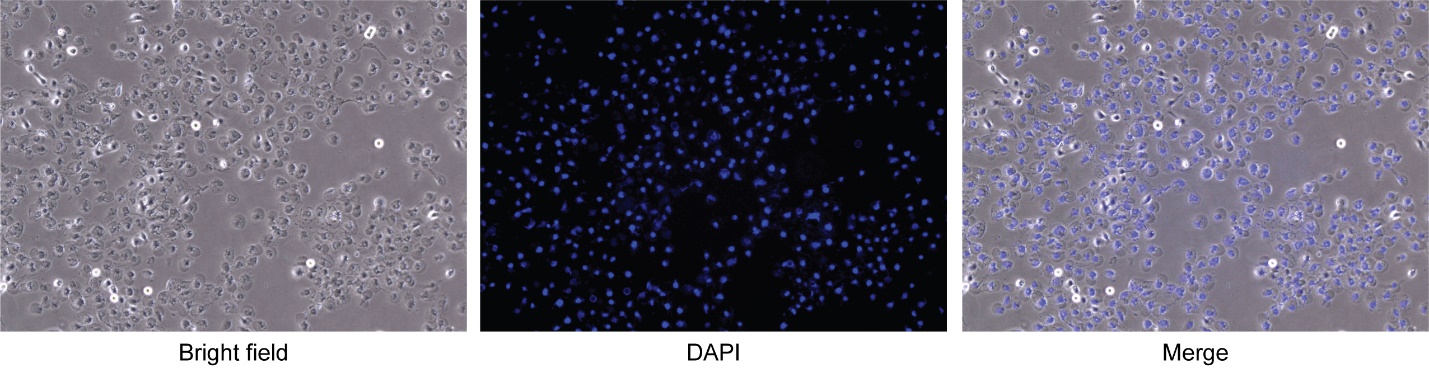
**

**Figure S3** Nuclear staining in HaCaT cells. To detect their nuclei, HaCaT cells were stained with DAPI and fluorescence was visualized with a fluorescence microscope. The blue color indicates the nucleus of each cell.


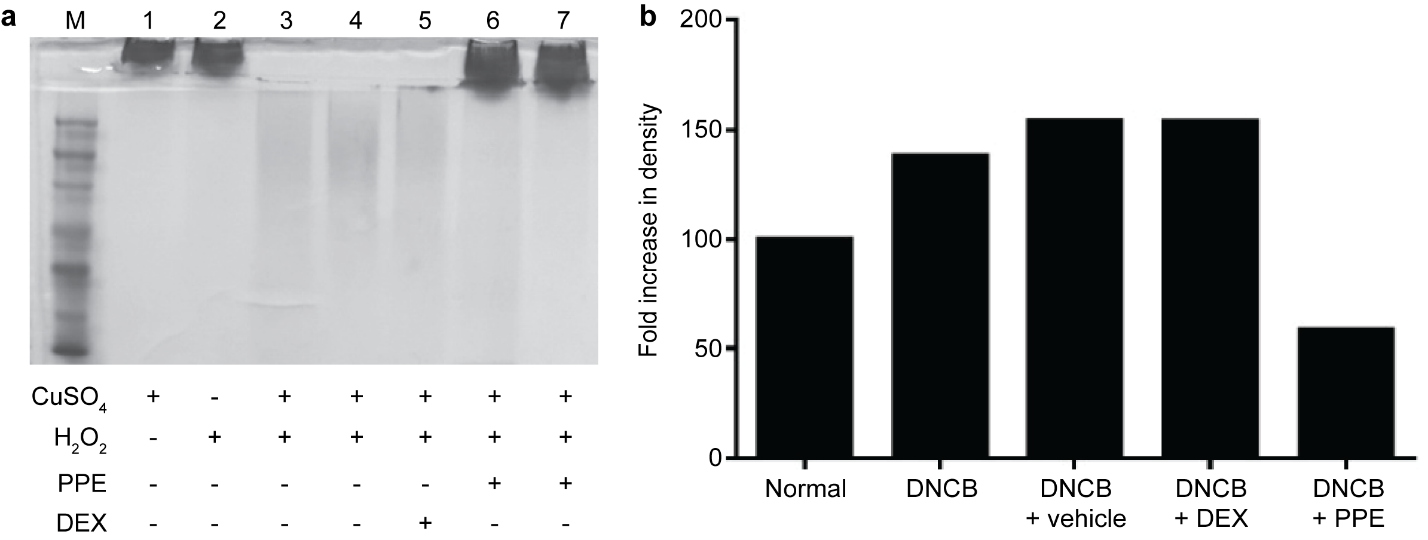


**Figure S4** PPE prevents the oxidative degradation of HA by inhibiting the hyaluronidase activity. (a) Analysis of the HA degradation by SDS-polyacrylamide gel electrophoresis. High molecular weight HA was incubated with ROS inducing agents (CuSO_4_ and/or H_2_O_2_) with or without the addition of different concentrations of PPE or DEX. Molecular weight of HA was analyzed by SDS-PAGE electrophoresis and HA was stained with 0.005% Stains all reagent. Each lane was treated as follows: 200 mM H_2_O_2_ in lane 3, 100 mM H_2_O_2_ in lane 4, 100 mM of DEX in lane 5, 0.1% PPE in lane 6, and 1% PPE in lane 7. (b) Inhibition of hyaluronidase activity by PPE treatment. Following the irritation of back skins of mice with DNCB patches, cream alone, DEX or PPE was topically applied. After sacrificing mice, 100 mg of back skins were collected and isolated tissue extracts. Total proteins obtained from tissue extracts were subjected to 6% SDS polyacrylamide gel containing high molecular weight HA and the gel was stained with Alcian blue to detect the hyaluronidase enzyme activity. The density of bands shown in gel represents the level of hyaluronidase activity and the fold increase density was plotted as a bar graph.

**Supplementary Methods**

**Detection of ROS-induced HA degradation *in vitro***

High molecular weight HA (Calbiochem, USA) was gently mixed with 0.1% PPE, 1% PPE or 100 mM DEX one day prior to the ROS induction. ROS were generated *in vitro* by the Cu(II)/H_2_O_2_ system as previously described (ref). Specifically, hydroxyl radicals and superoxide anions were produced by combining 50 µM CuSO_4_ with 100 or 200 mM H_2_O_2_ in 0.1 M NaH_2_PO_4_ buffer. Following the incubation of high molecular weight HA mixtures with ROS inducing agents for 1 h at room temperature, HA reactants were electrophoresed to 7% polyacrylamide gel and the molecular size of HA was visualized by staining a gel overnight with 0.005% Stains-All (Sigma-Aldrich, USA). After extensively destaining the gel with isopropyl alcohol, ROS-induced HA degradation was analyzed.

**Hyaluronic acid (HA) zymography**

Back skins of mice were stimulated with DNCB as described in Figure S1, and 100 mg of dorsal tissues were harvested on day 17. After collected tissues were lysed with RIPA buffer (Thermo Scientific, USA), the hyaluronidase activity was measured by Zymography as previously described (ref). Briefly, tissue lysates were subjected to electrophoresis on 6% SDS polyacrylamide gel containing 0.17 mg/ml of high molecular weight HA. Following electrophoresis, the gel was rinsed with buffer containing 50 mM HEPES and 3 % Triton X-100 and subsequently incubated in 0.15 M NaCl_2_/0.1 M Na-formate buffer for 18 h. The gel was then stained with 0.5% Alcian blue in 3% acetic acid, and the hyaluronidase activity was detected by analyzing the intensity of digested HA bands using a LAS-3000 imager (FujiFilm).
